# A Neodymium(III)-Based Hydrogen-Bonded Bilayer Framework with Dual Functions: Selective Ion Sensing and High Proton Conduction

**DOI:** 10.3390/molecules30173455

**Published:** 2025-08-22

**Authors:** Jie Liu, Xin-Yu Guo, Wen-Duo Zhu, Nan Zheng, Jiu-Fu Lu

**Affiliations:** College of Chemical and Environment Science, Shaanxi University of Technology, Hanzhong 723001, China; liujie@snut.edu.cn (J.L.); xinyuguo@snut.edu.cn (X.-Y.G.); zhengnan@snut.edu.cn (N.Z.)

**Keywords:** lanthanide hydrogen-bonded organic frameworks, luminescence properties, Hirshfeld surface analysis, fluorescence quenching, proton conductivity

## Abstract

Lanthanide hydrogen-bonded organic frameworks (Ln-HOFs) integrating luminescent and proton-conductive properties hold significant promise for multifunctional sensing and energy applications, yet their development remains challenging due to the difficulty of balancing structural stability and functional diversity. In this context, this study successfully synthesized a novel neodymium(III)-based hydrogen-bonded framework material, formulated as {Nd(H_2_O)_3_(4-CPCA)[H(4-CPCA)]∙H_2_O}ₙ (SNUT-15), via hydrothermal assembly using 1-(4-carboxyphenyl)-4-oxo-1,4-dihydropyridazine-3-carboxylic acid (H_2_(4-CPCA)) as the ligand. Single-crystal X-ray diffraction analysis revealed a rare two-dimensional hydrogen-bonded bilayer structure stabilized by π-π stacking interactions and intermolecular hydrogen bonds. Hirshfeld surface analysis further corroborated the structural characteristics of this material. Moreover, leveraging the superior luminescent properties of lanthanide elements, this crystalline material exhibits dual functionality: selective fluorescence quenching toward Fe^3+^, La^3+^, and Mn^2+^ (with detection limits of 1.74 × 10^−4^, 1.88 × 10^−4^, and 3.57 × 10^−4^ mol·L^−1^, respectively), as well as excellent proton conductivity reaching 7.92 × 10^−3^ S cm^−1^ under conditions of 98% relative humidity and 353 K (80 °C). As a multifunctional neodymium(III)-based HOF material, SNUT-15 demonstrates its potential for applications in environmental monitoring and solid-state electrolytes, providing valuable insights into the rational design of lanthanide-containing frameworks.

## 1. Introduction

Hydrogen-bonded organic frameworks (HOFs), a class of crystalline porous materials formed via the self-assembly of organic molecules or metal–organic units through intermolecular hydrogen bonds [1], have demonstrated significant potential in recent years due to their tunable structures, ease of functionalization, and unique physicochemical properties. These include applications in gas adsorption and separation, analytical monitoring, proton conduction, and luminescent materials [2,3,4,5]. Compared with metal-organic frameworks (MOFs), HOFs exhibit advantages such as remarkable stability, ease of in situ film formation, cost-effectiveness, and self-healing capabilities, making them particularly promising as solid-state electrolytes in fuel cells [6]. However, traditional HOFs are often limited by the relatively weak hydrogen bonding interactions, which result in challenges such as poor structural stability and difficulty in maintaining permanent porosity, thereby restricting their practical applications [7].

To overcome this bottleneck, current research emphasizes enhancing framework stability by constructing multiple hydrogen bond networks and incorporating secondary interactions (such as π-π stacking and van der Waals forces) in a synergistic manner [8]. Among them, the lanthanide elements serve as an ideal platform for designing multifunctional HOFs due to their excellent luminescent properties (sharp line emission, long fluorescence lifetime) and high coordination number [9]. Ln-HOFs are hybrid materials where Ln^3+^ ions are incorporated into the organic linker framework of the HOF skeleton via coordination or electrostatic interactions. As an important branch of functionalized HOFs, they exhibit diverse luminescent behaviors facilitated by the ligand-to-metal charge transfer-assisted energy transfer (LMCT-ET) process [10]. By introducing Ln^3+^ ions as functional reagents into the HOF matrix, a mixed system with multiple (primarily dual) luminescent centers is formed, enabling emission at different wavelengths within the visible spectrum. Ln-HOFs effectively integrate the intrinsic fluorescence of Ln^3+^ ions with the unique photoluminescence properties of HOFs, thereby yielding superior optical characteristics. Consequently, Ln-MOFs can be utilized as highly effective luminescent probes for detecting metal cations [11,12,13,14], anions [15,16], small molecules [17], explosives [18], and other analytes. However, Ln-HOFs that simultaneously possess dual functionalities of luminescent sensing and proton conduction remain scarcely reported, and the structural-performance regulation mechanism requires further exploration.

**Scheme 1:**
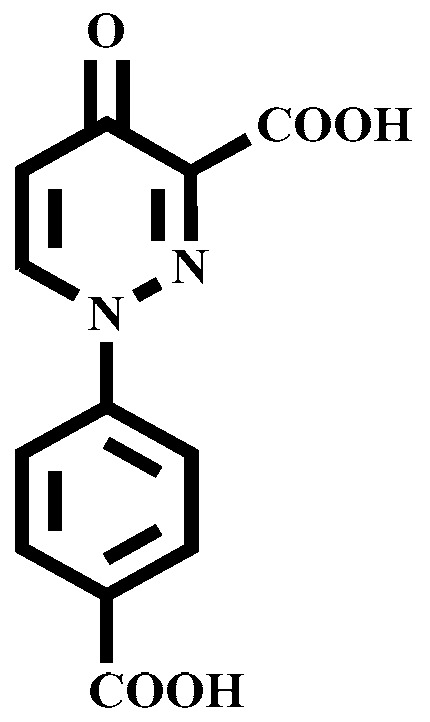
Structural diagram of H_2_(4-CPCA).

Therefore, via the hydrothermal assembly of the 1-(4-carboxyphenyl-4-oxadiazine)-3-carboxylic acid (H_2_(4-CPCA)) ligand (Scheme 1), this study successfully synthesized a novel neodymium(III)-based hydrogen-bonded framework material (SNUT-15). Single-crystal X-ray diffraction analysis revealed its rare two-dimensional hydrogen-bonded bilayer structure. Through intermolecular hydrogen bonding and π-π stacking interactions, the structure extends into a three-dimensional supramolecular network. Hirshfeld surface analysis confirmed that the dominant contributions of O-H (17.1%) and H-H (22.5%) interactions play a critical role in facilitating proton conduction. Moreover, owing to the excellent luminescent properties of lanthanide elements, this material exhibits dual functionalities: superior proton conductivity and fluorescence quenching performance. This work provides new insights into the design of lanthanide-based hydrogen-bonded framework materials for applications in environmental monitoring and solid-state electrolytes.

## 2. Results and Discussion

### 2.1. Crystal Structure Description of SNUT-15

**Figure 1:**
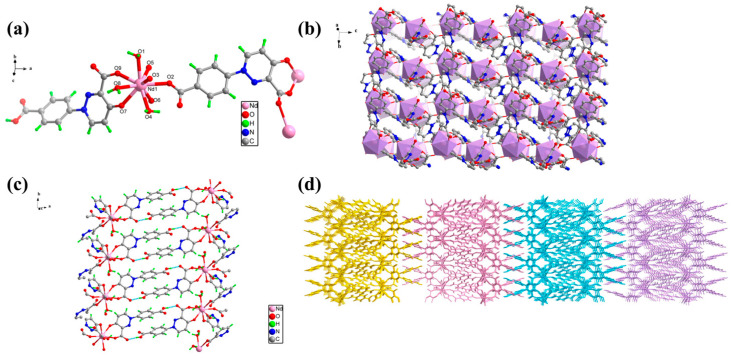
Crystal structure of SNUT-15: (**a**) Asymmetric unit; (**b**) Two-dimensional structure; (**c**) Hydrogen bonding network; (**d**) Three-dimensional supramolecular structure.

Single crystal X-ray analyses revealed that SNUT-15 crystallized in the monoclinic, C2/c space group. The asymmetric unit of SNUT-15 comprises one Nd^3+^ ion, one deprotonated H_2_(4-CPCA) molecule, one hydrogen-depleted H_2_(4-CPCA) molecule, three monodentate coordinated H_2_O molecules, and one free H_2_O molecule. As depicted in Figure 1a, the central Nd^3+^ ion is nine-coordinate, involving nine oxygen atoms. Specifically, four oxygen atoms (O5, O6, O7, O9) originate from the carboxylate and carbonyl groups of one chelating H_2_(4-CPCA) molecule, two oxygen atoms (O2, O3) are contributed by the carboxylate group of another chelating H_2_(4-CPCA) molecule, and three oxygen atoms (O1, O4, O8) stem from three monodentate coordinated water molecules. Along the a-axis, adjacent Nd^3+^ ions are bridged by the H_2_(4-CPCA) ligands, forming one-dimensional chains. These chains are further interconnected via the H_2_(4-CPCA) ligands to construct a two-dimensional double-layered structure (Figure 1b). Subsequently, extensive intermolecular hydrogen bonding interactions between the layers (Figure 1c) lead to the formation of a three-dimensional supramolecular architecture (Figure 1d).

### 2.2. FT-IR Spectra Analysis

**Figure 2:**
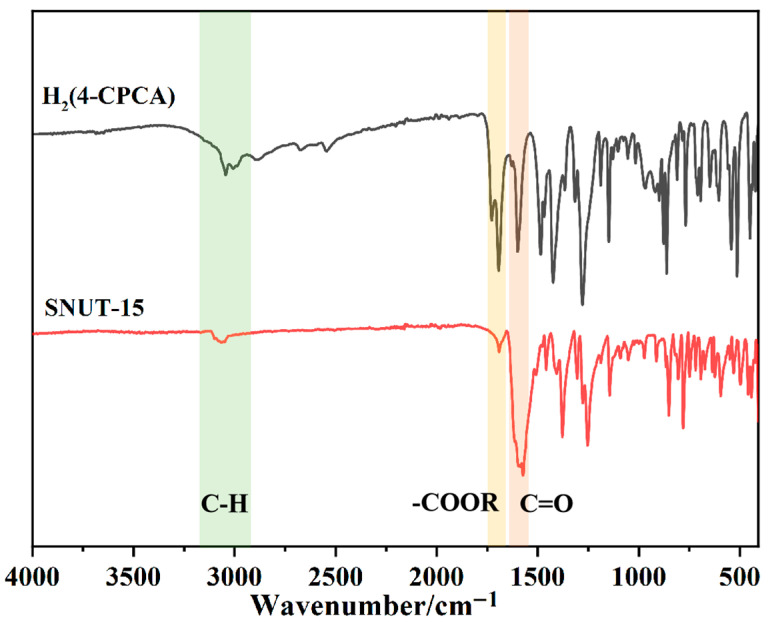
FT-IR spectra of SNUT-15.

The infrared spectroscopy analysis of SNUT-15 and H_2_(4-CPCA) was performed using the KBr pellet method, with a wavenumber range of 4000–400 cm^−1^. The results are presented in Figure 2. As shown in Figure 2, the peak at 1450 cm^−1^ corresponds to the bending vibration of C-H bonds in the benzene ring, while the stretching vibration of C-H bonds is observed at 2960 cm^−1^ [19]. The characteristic stretching vibration of the C=O group appears at 1655 cm^−1^. In H_2_(4-CPCA), the strong absorption band at 1601 cm^−1^ clearly indicates the presence of a C=N bond. The vibrational frequencies of C-N bonds within the N-heterocyclic ring of the ligand are distributed approximately between 640 cm^−1^ and 780 cm^−1^ [20]. Infrared spectroscopic analysis reveals a red shift in the C=O stretching vibration from 1655 cm^−1^ to 1640 cm^−1^, suggesting coordination between the oxygen atom of the ligand and the central metal ion Nd^3+^. This observation provides preliminary evidence for the successful synthesis of SNUT-15.

### 2.3. Powder X-Ray Diffraction Analysis

**Figure 3:**
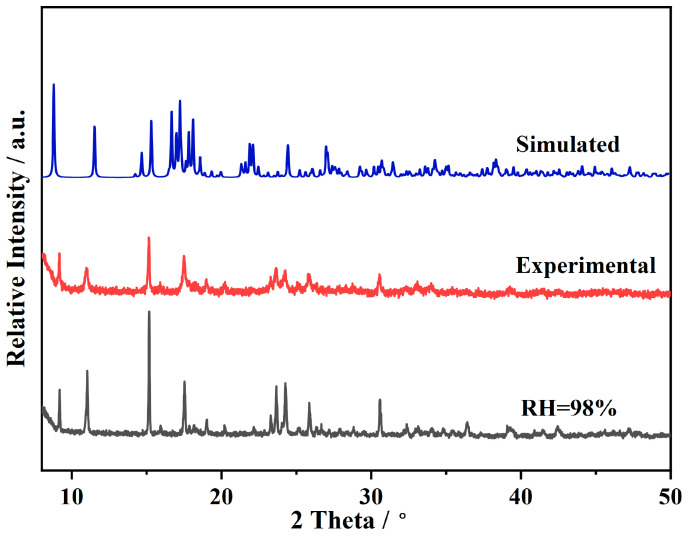
Powder X-ray diffraction pattern of SNUT-15.

SNUT-15 was characterized by powder X-ray diffraction (PXRD) analysis, and the resulting patterns were compared with the simulated spectra derived from the corresponding single crystal structure, as depicted in Figure 3. The experimental PXRD pattern aligns with the simulated profile, confirming phase purity. Minor peak shifts at lower angles (8° and 11°) are attributed to hydration-induced lattice expansion under ambient conditions—a hallmark of flexible H-bonded frameworks [21]. After 98% RH exposure, altered relative intensities at 12° and 17° arise from water-mediated reorientation of H-bonds, which enhances proton-conducting pathways without compromising crystallinity.

### 2.4. Thermal Analysis

**Figure 4:**
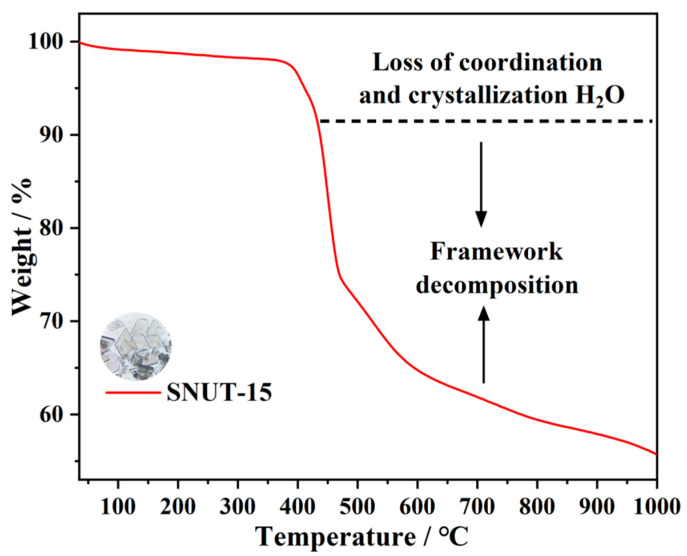
Thermogravimetric curve of SNUT-15.

To investigate the thermal stability of SNUT-15, thermogravimetric analysis (TGA) was carried out under a nitrogen atmosphere across a temperature range of 25–1000 °C, with a heating rate of 10 °C/min. As shown in Figure 4, SNUT-15 exhibited an 8.44% mass loss between 150 °C and 300 °C, which can be attributed to the desorption of free water molecules and three monodentate-coordinated water molecules. This indicates that the material maintains good thermal stability within this temperature range. A sharp weight loss observed above 400 °C is likely associated with the collapse of the entire framework structure. The mass loss between 400 °C and 480 °C corresponds to the decomposition of ligands monodentately coordinated to the central Nd^3+^ metal ion. Beyond 480 °C, further weight loss is attributed to the decomposition of ligands that are bridging-coordinated to the central Nd^3+^ ion.

### 2.5. Morphological Analysis

**Figure 5:**
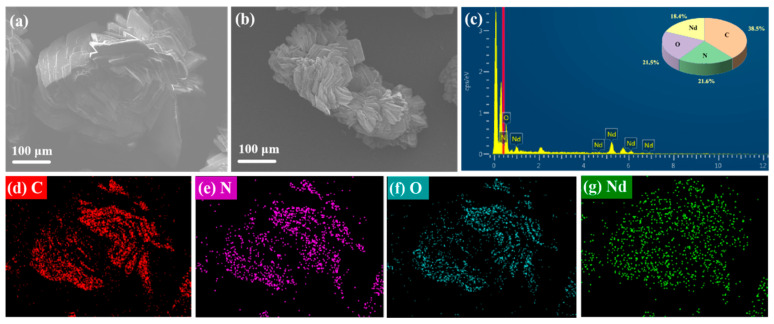
(**a**,**b**) show the SEM images of the SNUT-15 crystal morphology; (**c**) is the EDS image; (**d**–**g**) represent the distribution of C, N, O, and Nd elements in (**a**), respectively.

The morphology of SNUT-15 was observed using a scanning electron microscope (SEM). As depicted in Figure 5a,b, SNUT-15 exhibits single-dispersed, petal-like particles with a clearly visible layered arrangement. This characteristic arises from the presence of numerous hydrogen bonds within the structure of SNUT-15. The π-π stacking interactions between these hydrogen bonds lead to the formation of stable π-π coplanar aggregates, promoting layer-by-layer crystal growth. Additionally, SEM-EDS analysis was performed, as shown in Figure 5c. The results indicate that the crystal consists of four elements: C (38.5%), N (21.6%), O (21.5%), and Nd (18.4%). These elements are homogeneously distributed throughout the material, further confirming the successful synthesis of SNUT-15.

### 2.6. XPS Analysis

**Figure 6:**
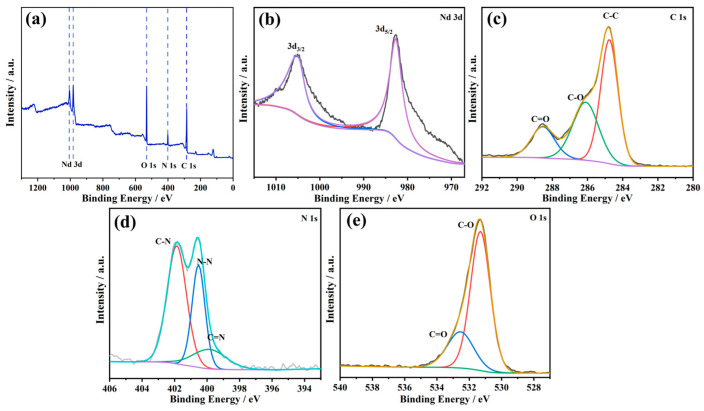
XPS spectral analysis of SNUT-15: (**a**) Full survey spectrum; (**b**) High-resolution spectrum of Nd 3d; (**c**) High-resolution spectrum of C 1s; (**d**) High-resolution spectrum of N 1s; (**e**) High-resolution spectrum of O 1s.

To further investigate the composition and structural characteristics of SNUT-15, X-ray photoelectron spectroscopy (XPS) was employed for material characterization. As shown in Figure 6. The full XPS survey spectrum presented in Figure 6a displays distinct characteristic peaks corresponding to Nd 3d, N 1s, C 1s, and O 1s, clearly confirming the presence of neodymium, nitrogen, carbon, and oxygen in the sample (Figure 6b). The high-resolution XPS spectrum of the Nd 3d region exhibits two prominent peaks at 982.9 eV and 1005.1 eV, corresponding to the Nd 3d_5/2_ and Nd 3d_3/2_ transitions, respectively, which are consistent with the binding energy values of Nd^3+^ ions [22]. In the C 1s spectrum (Figure 6c), a strong peak is observed at 284.8 eV, which can be assigned to the C–C bond, representing the dominant carbon species in SNUT-15. The peak at 286.1 eV corresponds to the C-O bond, indicating the presence of carbon-oxygen single bonds, while the peak at 288.6 eV is attributed to the C=O bond, confirming the existence of carbon-oxygen double bonds. These characteristic peaks collectively confirm the chemical state distribution of carbon in SNUT-15. The N 1s spectrum (Figure 6d) displays three distinct peaks at 401.9 eV, 400.5 eV, and 399.8 eV, which can be assigned to C-N, N-N, and C=N bonds, respectively, indicating that nitrogen primarily exists in the forms of carbon-nitrogen single bonds, nitrogen-nitrogen single bonds, and carbon-nitrogen double bonds. This further supports the presence of specific functional groups within SNUT-15. Additionally, in the O 1s spectrum (Figure 6e), the peak at 532.6 eV corresponds to the contribution of the C=O bond, while the peak at 531.4 eV is attributed to the C–O bond, confirming the presence of both carbon-oxygen double and single bond functionalities. Taken together, the XPS analysis provides strong evidence for the successful synthesis and structural integrity of SNUT-15.

### 2.7. Hirshfeld Surface Analysis

**Figure 7:**
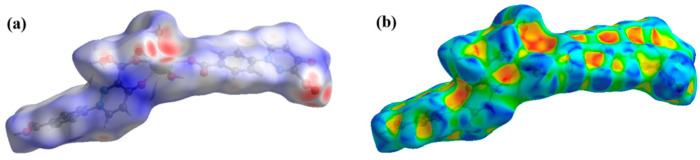
(**a**) d_norm_ plot of SNUT-15; (**b**) Shape index plot of SNUT-15.

**Figure 8:**
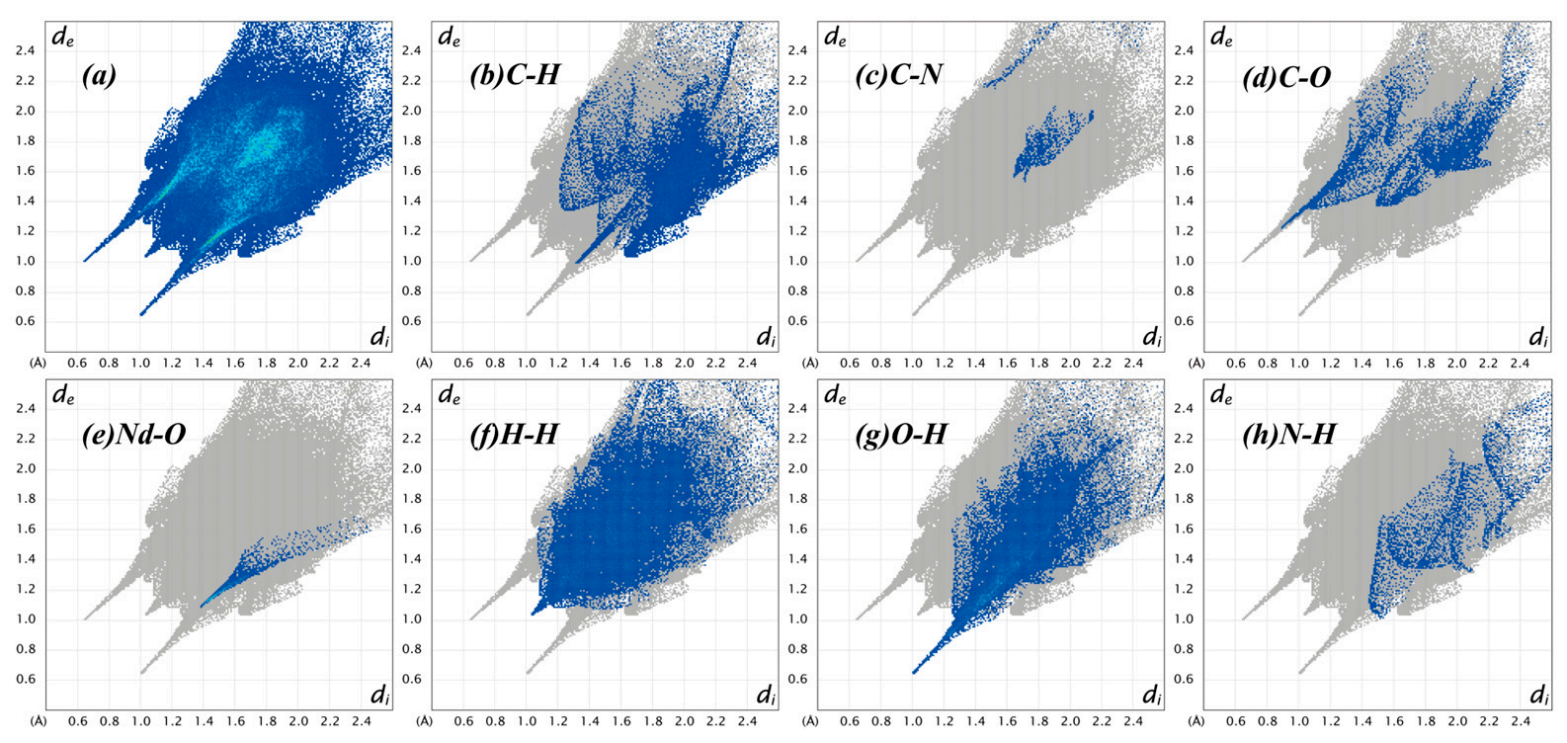
Two-dimensional fingerprint of SNUT-15; (**a**) all interactions, and delineated into; (**b**) C-H; (**c**) C-N; (**d**) C-O; (**e**) Nd-O; (**f**) H-H; (**g**) O-H; (**h**) N-H; and the di and de values are the closest internal and external distances (in Å) from given points on the Hirshfeld surface contacts.

From the single crystal structure analysis, it is evident that numerous intermolecular hydrogen bond interactions are critical for the formation of the three-dimensional supramolecular structure of SNUT-15 [23]. To further investigate the nature and proportions of these interactions, Hirshfeld surface and two-dimensional fingerprint analyses were performed using Crystal Explorer 17.5. The d_norm_ map of SNUT-15 is presented in Figure 7a, where the gray-white regions correspond to medium-strength hydrogen bond interactions (where molecular contacts equal the van der Waals radii), red regions indicate strong interactions, and blue regions represent weak or negligible interactions. The presence of significant short-range hydrogen bond interactions in SNUT-15 [24,25] can be inferred from the red regions in the d_norm_ map. The Shape index graph (Figure 7b) reveals intermolecular stacking effects, with blue and red-opposing areas indicating π-π stacking interactions within the complex. The two-dimensional fingerprint graph (Figure 8) illustrates the interatomic charge interactions between molecules in the complex, showing that H···H interactions account for 22.5%, while N-H, O-H, and C-H interactions contribute 2.3%, 17.1%, and 12.5%, respectively. These results demonstrate that O-H hydrogen bonds and H-H (π-π) interactions dominate and play a pivotal role in forming the three-dimensional supramolecular structure of SNUT-15 [26,27].

### 2.8. UV–Vis Absorption

**Figure 9:**
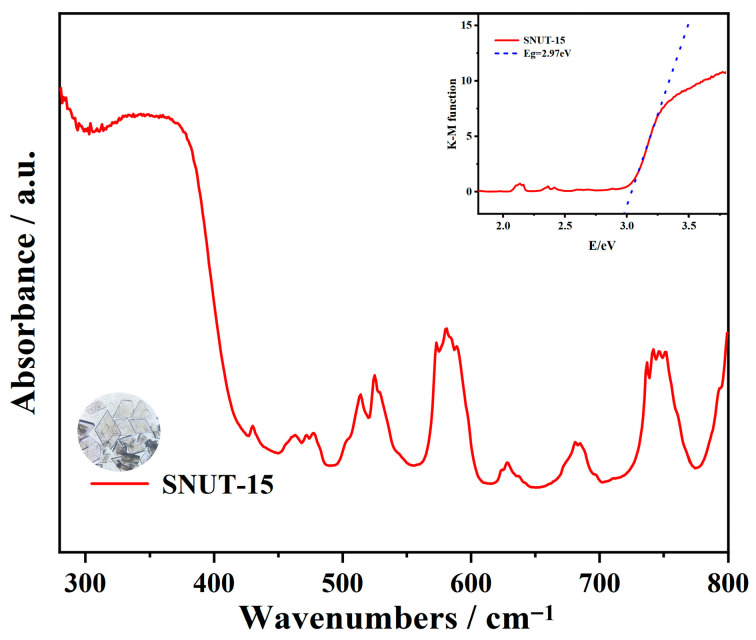
UV–vis DRS (diffuse reflection spectrum) and Kubelka–Munk-transformed DRS of SNUT-15.

The optical absorption properties of SNUT-15 were investigated using UV–Vis diffuse reflectance spectroscopy (UV–Vis DRS). The bandgap energy (Eg) was then determined based on the Kubelka–Munk (KM) model, expressed as α/S = (1 − R)^2^/2R, where α is the absorption coefficient, S is the scattering coefficient, and R denotes the reflectance. The calculated Eg value of SNUT-15 was found to be 2.97 eV, indicating a wide bandgap characteristic. This feature is particularly advantageous for potential applications in semiconductor materials (Figure 9).

### 2.9. Fluorescence Sensing Analysis

**Figure 10:**
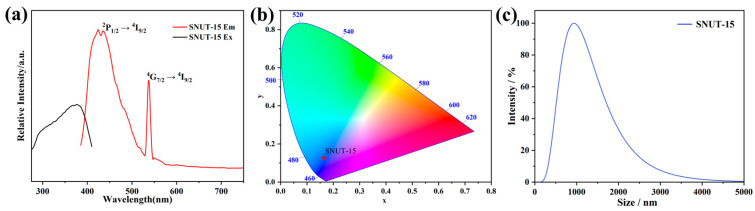
(**a**) Solid-state fluorescence spectrum of SNUT-15; (**b**) CIE chromaticity coordinate SNUT-15; (**c**) DLS measurement of SNUT-15.

In order to study the luminescent properties of SNUT-15, solid-state fluorescence analysis was performed, as depicted in Figure 10a. Under excitation at 378 nm, SNUT-15 exhibits a strong fluorescence emission peak at 424 nm, which is attributed to the ^2^P_1_/_2_ → ^4^I_9_/_2_ transition of Nd^3+^. A sharp emission peak at 537 nm corresponds to the ^4^G_7_/_2_ → ^4^I_9_/_2_ transition of Nd^3+^ [28]. The CIE 1931 chromaticity coordinates (Figure 10b) were calculated and determined to be (0.16, 0.12). Upon exposure to a UV lamp, SNUT-15 exhibited visible blue fluorescence, indicating its excellent fluorescence performance and potential as a blue light material. Based on these findings, fluorescence sensing studies were conducted on SNUT-15 to evaluate the changes in luminescence intensity induced by various metal ions. The results demonstrated that the addition of Fe^3+^, La^3+^, and Mn^2+^ ions led to fluorescence quenching effects. The DLS test was performed on SNUT-15, with the results presented in Figure 10c. Dynamic light scattering (DLS) analysis revealed that the particle size of SNUT-15 was approximately 936 nm. The particle size distribution displayed a distinct single peak, indicating that the sample remained well-dispersed in the medium without significant agglomeration. This observation confirms the material’s excellent dispersibility and stability in the solution system.

**Figure 11:**
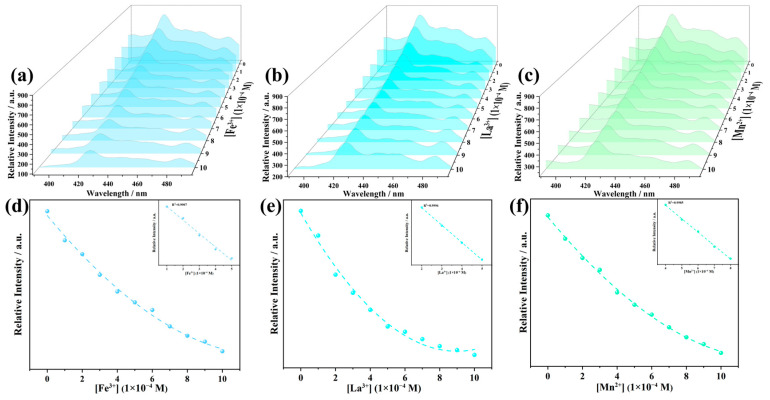
Luminescence intensity of SNUT-15 in solutions with different concentrations of Fe^3+^, La^3+^, and Mn^2+^ and their fitting values; (**a**–**c**) represent the luminescence intensity of SNUT-15 after adding solutions with different concentrations of Fe^3+^, La^3+^, and Mn^2+^ respectively; (**d**–**f**) are the linear fittings of the fluorescence change values of SNUT-15 in Fe^3+^, La^3+^, and Mn^2+^.

To investigate the capability of SNUT-15 as a fluorescent probe for detecting Fe^3+^, La^3+^, and Mn^2+^, solutions of SNUT-15 were prepared, and Fe^3+^, La^3+^, and Mn^2+^ ions were added in a stepwise gradient manner. The fluorescence responses were subsequently recorded, as illustrated in Figure 11a–c. Upon increasing the concentrations of Fe^3+^, La^3+^, and Mn^2+^, the fluorescence emission intensity of SNUT-15 exhibited a gradual quenching effect. Furthermore, as depicted in Figure 11d–f, within a specific concentration range, the fluorescence intensity of SNUT-15 demonstrated an excellent linear correlation with the concentrations of Fe^3+^, La^3+^, and Mn^2+^ (Fe^3+^: R^2^ = 0.9907, La^3+^: R^2^ = 0.9996, Mn^2+^: R^2^ = 0.9985).

The detection limit (LOD) serves as a critical parameter for evaluating the sensitivity of fluorescence-based detection. It can be calculated using the formula LOD = 3σ/K_sv_, where σ represents the standard deviation of the fluorescence intensity obtained from 11 blank experiments conducted without any added ions for the tested complex, and K_sv_ is the Stern-Volmer quenching constant [29]. Based on this formula, the detection limits of SNUT-15 for Fe^3+^, La^3+^, and Mn^2+^ were determined to be 1.74 × 10^−4^ mol·L^−1^, 1.88 × 10^−4^ mol·L^−1^, and 3.57 × 10^−4^ mol·L^−1^ respectively. These results indicate that SNUT-15 exhibits a low detection limit and a broad linear detection range, highlighting its potential as an effective fluorescent probe.

**Figure 12:**
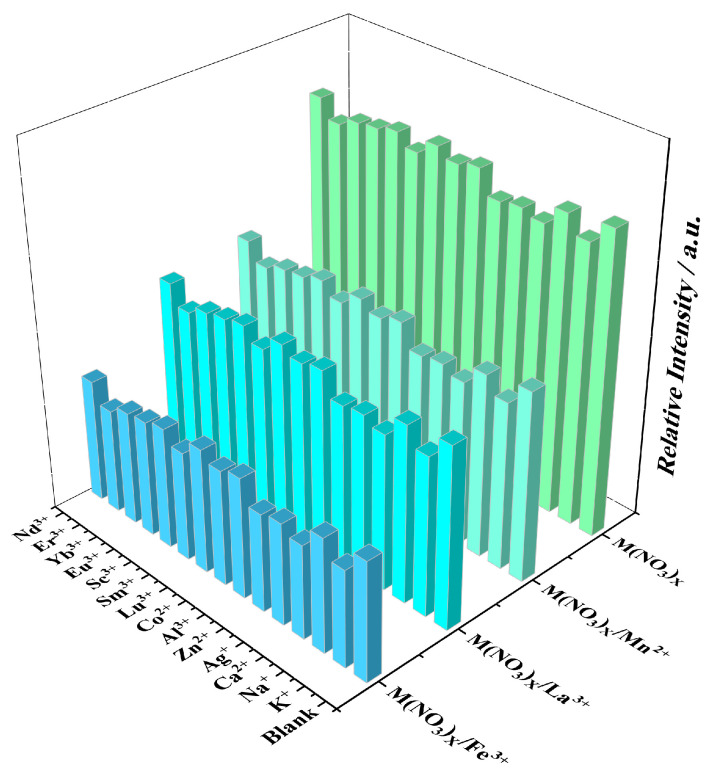
Anti-interference diagram of SNUT-15.

To further investigate the selective detection capability of SNUT-15 toward Fe^3+^, La^3+^, and Mn^2+^, a series of metal ions (Na^+^, K^+^, Cd^2+^, Zn^2+^, Ca^2+^, Co^2+^, Ag^+^, Al^3+^, Cu^2+^, Eu^3+^, Sc^3+^, Nd^3+^, Er^3+^, Yb^3+^, Sm^3+^, Lu^3+^) at a concentration of 0.1 mmol/L were introduced into the SNUT-15 suspension. Following ultrasonic treatment to ensure the formation of a stable suspension, additional SNUT-15 was added. Under room temperature conditions, the fluorescence emission spectra were recorded with an excitation wavelength of 378 nm. As shown in Figure 12, it is evident that even in the presence of various interfering metal ions, SNUT-15 exhibits high selectivity for Fe^3+^, La^3+^, and Mn^2+^.

### 2.10. Proton Conductivity Analysis

**Figure 13:**
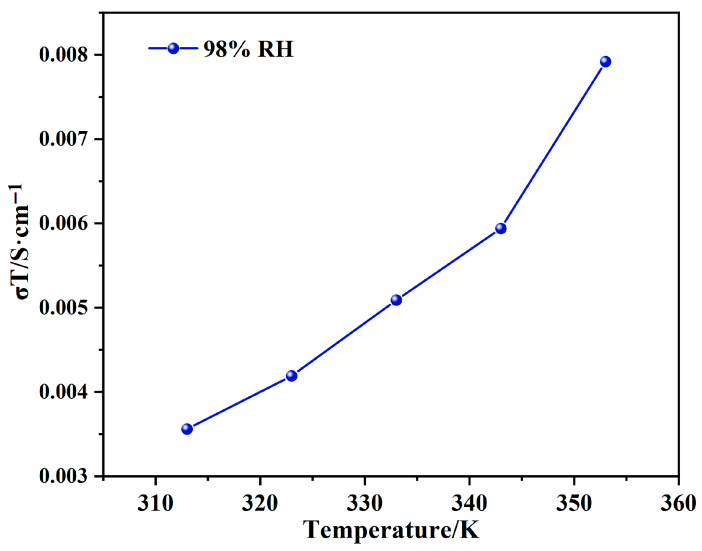
Conductivity of SNUT-15 at 313 K-353 K and 98% RH.

**Figure 14:**
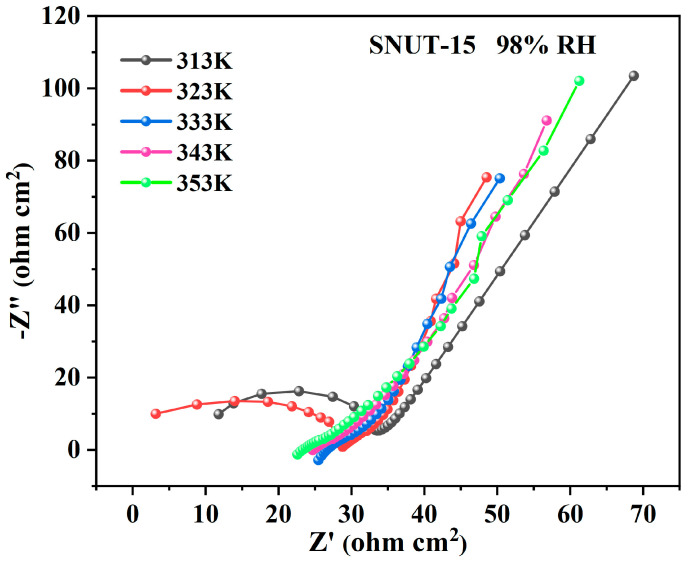
Nyquist plots of SNUT-15 at 98% RH.

The proton conductivity of SNUT-15 was measured via impedance spectroscopy within the temperature range of 313 K to 353 K and at a relative humidity of 98%. The results are presented in Figure 13. At constant relative humidity, the proton conductivity of SNUT-15 exhibits a positive correlation with temperature. Specifically, at 98% RH, the proton conductivity (σ) of SNUT-15 increased from 3.56 × 10^−3^ S cm^−1^ at 313 K to 7.92 × 10^−3^ S cm^−1^ at 353 K. This enhancement in proton conductivity can be primarily attributed to the increase in temperature, which not only provides the necessary energy for proton dissociation but also accelerates the migration of proton carriers, thereby promoting proton transfer. As shown in Figure 14, the Nyquist plots of the material at different temperatures exhibit high similarity. With increasing temperature and humidity, the radius of the semicircle gradually decreases, indicating an increase in proton conductivity.

**Figure 15:**
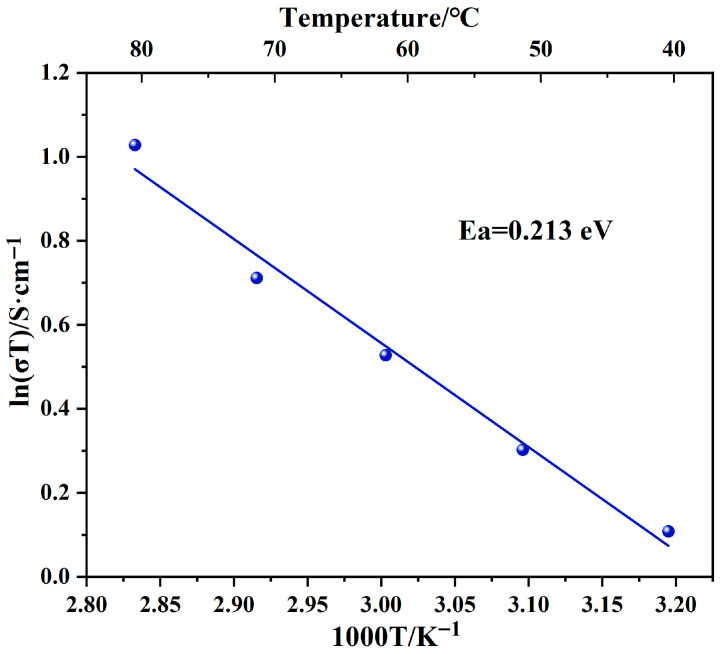
Arrhenius plots of proton conductivity of 98% RH.

The activation energy of SNUT-15 is presented in Figure 15. Studies have shown that the proton transport mechanisms in solid conductors can primarily be categorized into two types: the Grotthuss mechanism and the Vehicle mechanism [30,31,32]. The most significant distinction between these two mechanisms lies in their activation energy requirements. The Grotthuss mechanism operates with a lower activation energy (Ea < 0.4 eV) and relies predominantly on the hydrogen bond network formed by the material for proton conduction. In contrast, the Vehicle mechanism involves the diffusion of proton-bound carriers in a specific direction, while the remaining carriers diffuse in the opposite direction. The proton conductivity in this mechanism can be enhanced by increasing the concentration of proton carriers, which typically requires a higher activation energy (Ea > 0.4 eV). Within the range of 98% relative humidity, the activation energy Ea value of SNUT-15 is determined to be 0.213 eV, indicating that its proton conduction follows the Grotthuss mechanism.

## 3. Experimental Section

### 3.1. Materials and Methods

The H_2_(4-CPCA) ligand was synthesized in accordance with our reported method [23,33]; NdCl_3_·6H_2_O, obtained from Jinan Henghua Technology Co. (Jinan, China); 1,10-Phenanthroline (phen), supplied by Shanghai Haohong Biomedical Technology Co. (Shanghai, China); Sodium hydroxide (NaOH), purchased from Tianjin Kemiou Chemical Reagent Co. (Tianjin, China).

The infrared spectra (IR) were recorded on a Bruker Equinox-55 FTIR spectrometer (Bruker, Ettlingen, Germany) within the range of 4000–400 cm^−1^ using KBr pellets. Elemental analysis (EA) was performed with a Vario EL III elemental analyzer (Elementar Analysensysteme GmbH, Langenselbold, Germany). Crystallographic data were collected on a Bruker APEX-II CCD X-ray diffractometer (Bruker, Germany). Powder X-ray diffraction (PXRD) measurements were conducted on a Bruker D8 Advance X-ray diffractometer (Bruker, Germany). Simulated PXRD patterns were generated using the Mercury 3.1 software package. Thermogravimetric analysis (TGA) was carried out on an SDT Q600 thermal analyzer (TA Instruments, New Castle, DE, USA) under a nitrogen atmosphere, over a temperature range of 25–1000 °C at a heating rate of 10 °C/min. UV–Vis absorption spectra were acquired using a UV-2600 spectrophotometer (Shimadzu, Kyoto, Japan).

### 3.2. Synthesis of {Nd(H_2_O)_3_(4-CPCA)[H(4-CPCA)]∙H_2_O}_n_ (SNUT-15)

A mixture of NdCl_3_·6H_2_O (0.1 mmol), H_2_(4-CPCA) (0.05 mmol) and phen (0.05 mmol) was dissolved in H_2_O (2 mL), the pH of the resulting solution was adjusted to 5.2 using a 0.3 mol/L sodium hydroxide solution. Mixed solvent was placed in a 25 mL glass scintillation vial, under heated to 110 °C for 48h, and then collect the crystalline product and wash it three times with deionized water. Subsequently, vacuum filtration was performed. The filtered solid was then placed in a vacuum oven at 30 °C and dried under constant temperature conditions for 3 h. Pale purple block-shaped crystals were obtained as the final product, with a yield of 84.2% (based on Nd). Elemental analysis (%): Calcd for C_24_H_21_N_4_NdO_14_ (Mr = 733.69): C 39.29, H 2.88, N 7.64; found: C 39.25, H 2.86, N 7.63. IR (cm^−1^): 2960(m), 1655(s), 1601(s), 1450(m), 774(m).

### 3.3. Determination of Crystal Structures

A crystal with dimensions of 0.20 mm × 0.11 mm × 0.05 mm was selected for X-ray diffraction experiments conducted at room temperature. The diffraction data were collected using a Bruker-ApexП X-ray single crystal diffractometer equipped with monochromatized Mo-Kα radiation (λ = 0.71073 Å) and a graphite monochromator. Data acquisition was performed in the ω-2θ scanning mode, and all measurements were corrected for Lorentz and empirical absorption effects. The crystal structure was determined by direct methods utilizing appropriate software, while hydrogen atoms were located through difference Fourier synthesis and refined to their calculated optimal positions. The SHELX-97 program (Use Olex2 1.5) facilitated the refinement of all non-hydrogen atoms along with their anisotropic thermal parameters via full-matrix least-squares methods [34,35]. Crystallographic data of structural analyses for molecular was summarized in Table 1. The main bond lengths and angles of SNUT-15 and are listed in Table 2.

**Figure 16:**
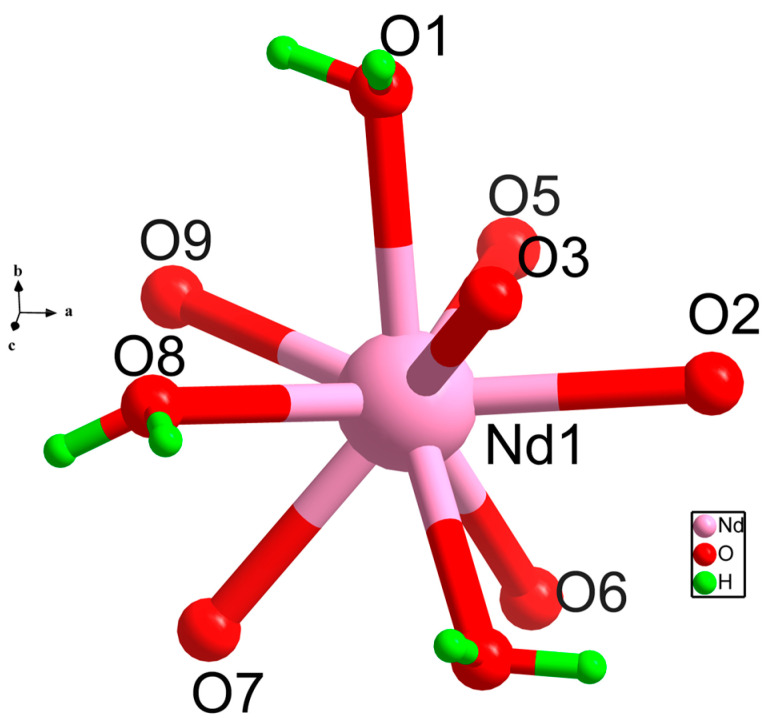
Coordination environment diagram of Nd^3+^.

### 3.4. Fluorescence Sensing Measurements

The powder samples of SNUT-15 (2 mg) were immersed in 4 mL DEA, which was then ultrasonicated for 20 min to obtain a stable suspension. The corresponding metal ion salts were accurately weighed using an analytical balance and subsequently dissolved in deionized water to prepare 0.1 mol/L aqueous solutions of Na^+^, K^+^, Pb^2+^, Cd^2+^, Zn^2+^, Mn^2+^, Ba^2+^, Hg^2+^, Ca^2+^, Co^2+^, Ag^+^, Ni^2+^, Cu^2+^, Eu^3+^, Sm^3+^, Nd^3+^, Gd^3+^, Pr^3+^, Dy^3+^, La^3+^, Ho^3+^, and Lu^3+^ at a concentration of 0.1 mol/L, as well as tetracycline, ascorbic acid, and dopamine at a concentration of 0.01 mol/L were prepared using the aforementioned method for qualitative analysis and anti-interference studies. For quantitative titration studies, 3 μL of each prepared suspension was added per trial. The fluorescence spectra were recorded under excitation at a wavelength of 378 nm.

### 3.5. Proton Conductivity

The dried SNUT-15 material (30–50 mg) was pressed at a pressure of 0.8 MPa to form circular disks with a diameter of 6 mm. The thickness of the disks was measured using a vernier caliper. One side of each disk was coated with conductive silver adhesive and subsequently fixed onto a glassy carbon electrode. A beaker equipped with a high-temperature circulating water bath was employed to precisely control the temperature. Prior to the initial activation test, the sample was immersed in the beaker for 24 h. Whenever the temperature was altered, the sample was allowed to equilibrate for at least 1 h to ensure stable temperature and relative humidity conditions. The proton conductivity (σ, S cm^−1^) of the sample was calculated using the following formula:

σ = L/(RS)(1)

ln (σ T) = ln A − Ea/(k_B_ T) (2)

σ denotes the electrical conductivity (S cm^−1^), L is the thickness of the tablet sample (cm), S represents the cross-sectional area of the tablet sample (cm^2^), R is the resistance value obtained from the test (Ω), A is the prefactor, k_B_ is the Boltzmann constant, and Ea is the activation energy.

## 4. Conclusions

In conclusion, this study successfully designed and synthesized a novel neodymium(III)-based hydrogen-bonded framework material (SNUT-15). Single-crystal X-ray diffraction analysis revealed a rare two-dimensional hydrogen-bonded bilayer structure. Furthermore, the successful synthesis and morphology of SNUT-15 were confirmed through powder XRD, FTIR, thermal analysis, SEM, and EDS characterizations. The results demonstrated that SNUT-15 not only exhibited selective fluorescence quenching toward Fe^3+^, La^3+^, and Mn^2+^ but also possessed an extensive hydrogen-bonding network that endowed it with excellent proton-conduction properties. Under conditions of 98% relative humidity and 80 °C, SNUT-15 displayed remarkable proton conduction performance. As a multifunctional neodymium(III)-based HOF material, this work provides valuable insights into the design of lanthanide-containing frameworks for applications in environmental monitoring and solid-state electrolytes.

## Figures and Tables

**Table 1:** Crystallographic data of SNUT-15.

Molecular Formula	C_24_H_21_N_4_NdO_14_
CCDC number	2,463,804
Relative molecular mass	733.69
T/K	293.00
Crystal system	monoclinic
Space group	C2/c
a/Å	60.631(13)
b/Å	7.3249(14)
c/Å	11.959(2)
α/(°)	90
β/(°)	94.262(8)
γ/(°)	90
Volume/Å^3^	5296.7(18)
Z	8
F(000)	2920.0
Crystal size/mm^3^	0.2 mm × 0.11 mm × 0.05 mm
2θ range for data collection/°	5.39°~51.314°
Index ranges	−73 ≤ h ≤ 73, −8 ≤ k ≤ 8, −14 ≤ l ≤ 12
Final R indices [I > 2σ(I)]	R_1_ = 0.0260, wR_2_ = 0.0684
Final R indexes [all data]	R_1_ = 0.0329, wR_2_ = 0.0717

**Table 2:** Selected bond lengths (Å) and angles (°) for SNUT-15 (Figure 16).

Bond	Bond Length/Å	Bond	Bond Angle/(°)
Nd1-O4	2.440(2)	O4-Nd1-O3	76.30(7)
Nd1-O3	2.521(2)	O4-Nd1-O2	133.52(8)
Nd1-O2	2.486(2)	O4-Nd1-O1	141.25(8)
Nd1-O1	2.461(2)	O4-Nd1-O5	90.85(8)
Nd1-O5	2.505(2)	O2-Nd1-O3	71.97(7)
Nd1-O13	2.551(2)	O2-Nd1-O5	135.17(8)

## Data Availability

The data presented in this study are available in the article. The datasets used and/or analyzed during the current study are available from the corresponding author on reasonable request.
